# ACTH (Acthar Gel) Reduces Toxic SOD1 Protein Linked to Amyotrophic Lateral Sclerosis in Transgenic Mice: A Novel Observation

**DOI:** 10.1371/journal.pone.0125638

**Published:** 2015-05-08

**Authors:** Hasan Arrat, Thomas J. Lukas, Teepu Siddique

**Affiliations:** 1 Department of Neurology, Northwestern University, Feinberg School of Medicine, Chicago, IL, 60611, United States of America; 2 Department of Pharmacology, Northwestern University, Feinberg School of Medicine, Chicago, IL, 60611, United States of America; University of Florida, UNITED STATES

## Abstract

Amyotrophic lateral sclerosis (ALS) is a neurodegenerative disease with a complex etiology and pathology that makes the development of new therapies difficult. ACTH has neurotrophic and myotrophic effects, but has not been tested in an ALS mouse model. The G93A-SOD1 mouse model of ALS was used to test the ability of this drug to delay ALS-like symptoms. We showed that within a specific dose range, ACTH significantly postponed the disease onset and paralysis in the mouse model. To our surprise and of greater significance is that ACTH significantly reduced the levels of soluble SOD1 in the spinal cord and CNS tissues of G93A-SOD1 treated mice as well as cultured fibroblasts.

## Introduction

Neurodegenerative diseases are a challenging group of central nervous system disorders for which there is no effective treatment or cure. Most of these diseases are fatal in the majority of affected patients, especially in the case of amyotrophic lateral sclerosis (ALS), where survival after diagnosis is less than 5 years. Approximately 90% of ALS cases occur in patients with no prior family history and are referred to as sporadic ALS (SALS) [1]. The remaining 10% of cases have heterogeneously genetic etiologies inherited and are called familial ALS (FALS) [2]. The first discovered cause of FALS is due to the mutations in gene which encodes the cytosolic Cu,Zn- super oxide dismutase (SOD1) enzyme [3,4]. Eight percent of ALS cases without a family history, either have mutations in *SOD1* or have an intronic expanded hexanucleotide (GGGGCC) repeat in *C9ORF72* [5,6]. In both familial and sporadic cases, large motor neurons of the spinal cord, cerebral cortex, and brain stem degenerate and cause progressive wasting and paralysis of voluntary muscles. Pathological changes in surviving motor neurons include simplification of the dendritic tree and loss of synaptic integrity, chromatolysis, hyaline inclusions, and accumulation of neurofilaments, ubiquitinated protein products, and Bunina bodies. Mutations in other genes can cause ALS at lower frequencies and include *FUS* [7], TDP43/*TARDBP* [8,9], optineurin/*OPTN* [10], Profilin/*PFN1* [11], *SQSTM1* [12,13] and ubiquilin2/*UBQLN2* [14]. In addition, various syndromes of motor neuron degeneration are caused by mutations in other genes such as Progranulin/*GRN*[15], Angiogenin/*ANG* [16], *CHMP2B*[17], Spastin/*SPAST*[18], *PRPH* [19], *VAPB* [20], and Alsin/*ALS2* [21]. Interestingly, Ubqln2 is found in inclusions or aggregates in nearly all cases of ALS [14]. Mutations in genes such as *FUS*, *TARBP*, *UBQLN2*, and *SQSTM1* can also cause dementia concurrent with ALS or without ALS.

The G93A-SOD1 mutation was the first ALS gene to be modeled in a transgenic mouse [22]. The model contains multiple copies of the entire human G93A-SOD1 gene and its promoter. This transgenic mouse model is the most frequently used model proximate to human ALS for testing potential therapies for human ALS and pathologic research experiments. There is debate about the relevance of the G93A-SOD1 transgenic mouse as a universal ALS model [23,24], however, it has been shown that the G93A-SOD1 mouse model (especially the lower expressing strain) has many pathological and clinical features in common with human FALS [25]. Furthermore, recent data has shown similarities between human G93A SOD1 and SALS in clinical course as well as in electrophysiological and pathology studies [26]. Many preclinical studies have been done on the G93A-SOD1 mouse model using different therapeutic approaches, but most candidates have failed in human clinical trials [23]. Riluzole, which was originally thought to work via the glutamate pathway [27], is the only FDA approved drug. In cultured motor neurons from the G93A-SOD1 mouse Riluzole was found to affect sodium channel currents at physiological concentrations [28]. One part of the mechanism may be through Riluzole-mediated inhibition of protein kinase C phosphorylation events that modulate channel activities [29]. On account of the multietiologic genetics of ALS, it has become clear that an etiology-driven approach focused on reducing the expression of toxic protein may be a more rational and effective therapy. Further, such an approach modeled in genetically engineered mice and found to be efficacious, may only be so in etiologically specific cases of ALS.

Acthar is a long-acting adrenocorticotropin (ACTH 1–39 in 16% gelatin). ACTH is one of several products driven by the POMC gene resulting in a polypeptide precursor containing 241 amino acid residues. The encoded protein undergoes post-translational enzymatic processing and cleavage by tissue-specific prohormone convertases. The anterior lobe of the pituitary gland is the essential site of this protein’s synthesis, during which adrenocorticotropin (ACTH) and β-lipotropin are the main end products. It has been known for decades that there are reciprocal actions between pituitary neuropeptides and proinflammatory cytokines [30]. It has also been shown that the application of an ACTH analog in both pre- [31] and post-symptomatic stages of experimental allergic neuritis [32] have ameliorated the impaired functional and histological parameters. This is relevant because of the alleged involvement of oligodendrocytes in ALS pathology [33]. ACTH analogs influence neuromuscular function by electrophysiological enhancement of motoneurons as well [34]. This same ACTH analog exhibits both neurotrophic [35] and myotrophic [36] properties.

Whether or not ACTH can be used as a therapy for neurodegenerative diseases had not been specifically tested when we conducted these experiments. Now an Acthar treatment protocol is underway in ALS patients (NCT01906658). Both intramuscular (IM) and subcutaneous (SC) administration of Acthar gel have been shown to have bio-equivalent effects on the cortisol stimulation response [37]. Given these positive neurotrophic activities, we tested the possible impact of Acthar gel on the G93A-SOD1 transgenic mouse model using both IM and SC routes of administration.

## Materials and Methods

### Animals

We used G93A transgenic mice expressing a high copy number of human SOD1 with a G93A mutation, which develop ALS-like symptoms and pathology [22,38,39]. This mouse line was held in our laboratory by breeding G93A males with B6sjl females purchased from Jackson Laboratory. The mutant offspring were identified by testing tail DNA using PCR.

Ethics Statement: This study was carried out in strict accordance with the recommendations in the Guide for the Care and Use of Laboratory Animals of the National Institutes of Health. The protocol (# 2009–1823) was approved by the Animal Care and Use Committee of Northwestern University.

### Experimental design

In this study we used a total number of 70 mice, divided into one control and four treatment groups, with each group consisting of half males and half females. Test and control animals were randomly selected from several litters of G93A-SOD1 positive animals. The control group (n = 16) was given IM 5% gelatin (Sigma), group two (n = 14) was given 120 U/kg Acthar gel IM every other day, group three (n = 18) was given 120 U/kg SC every other day, group four (n = 14) was given 60 U/kg SC every other day and group five (n = 10) was given 60 U/kg SC weekly, as summarized in Table 1. Treatment was started at 60 days of age, during the presymptomatic period of G93A mouse model. The treatment was continued until the end stage, which was determined clinically.

**Table 1:** Experimental treatment arms for the study.

Males					Females				
**Control**	**IM120**	**SC120**	**SC60**	**SC60-W**	**Control**	**IM120**	**SC120**	**SC60**	**SC60-W**
**8**	**7**	**9**	**6**	**5**	**8**	**7**	**9**	**8**	**5**

IM: intramuscular injection, SC: subcutaneous injection.

### Body weight and motor performance

A Model 7650 Rotarod was used to assess the motor coordination and fatigue resistance of mice; mice were adapted to the equipment for three days before data collection began. Mice were placed on Rotarod rotating at 15 rpm and timed until they fell off. Time was recorded in seconds three times weekly and averages were calculated for each individual mouse. Body weight was measured after each Rotarod performance. Rotarod measurements were conducted by the same individual who administered the drug. A second individual who was blind to the treatment was involved in the clinical assessments.

### Clinical assessments

Disease onset was defined by the date of the tremors, seen clinically by the tremor of the hind limbs when the mouse is suspended in the air by the tail and skin of the back. A mouse is considered paralyzed when it starts dragging its foot while walking, or has a drop foot when it is suspended on its back. The end stage was defined as when the mouse is no longer able to right itself within 30 seconds when it is placed down on its side.

### Biochemical changes of SOD1 protein in G93A SOD1 mice

The protein expression in different CNS parts was detected by ELISA. At the end stage, the mice were perfused by PBS and the brains and spinal cords were collected and frozen at -80°C. Tissues were homogenized in M-PER (Thermo Scientific, 78501) containing protease inhibitor (Complete Mini, 11836153001) one tablet in 10ml. After centrifugation for 15,000 rpm for 90 minutes at 4°C the supernatant was subjected to SOD1 ELISA measurement and GAPDH activity.

### Effect of ACTH on SOD1 levels in cultured G93A mouse fibroblasts

Fibroblasts from G93H mice were obtained and cultured as described previously [40]. ACTH (1–39) peptide (Rat—Sigma-Aldrich A7075) was added in media to cells seeded in 6 well plates at 300,000 cells/well. After 48 hr cells were washed with phosphate-buffered saline and lysed with M-PER reagent using three freeze-thaw cycles. Extracts were centrifuged at 15000 rpm for 1 hr and supernatants stored at -80°C. Extracts were assayed for SOD1 by ELISA and GAPDH activity for normalization.

### SOD1 ELISA assay

96-well assay plates were coated with sheep polyclonal anti-human-SOD1 antibody (Abcam) 1:200 dilution in 1x carbonate buffer. After blocking with 3% Bovine Serum Albumin in 1x PBS, 10 μL of cell lysates diluted to 100 μL in PBS were analyzed in triplicate. Rabbit monoclonal anti-SOD1 (Sigma) and HRP-conjugated goat anti-rabbit were sequentially added to the wells with incubation at 37°C for one hour each. Wells were washed three times with PBS-0.01% Tween 20 between each step. Color was developed by adding substrate solution (Sigma Fast o-phenylenediamine dihydrochloride tablet in 20ml of water) for 10 minutes in the dark. The reaction was stopped by adding 3M HCl and the absorbance read at 485 nm. Standard curves were prepared from human erythrocyte SOD1 (Sigma) on the same plate.

### GAPDH activity

To measure GAPDH activity we used the KDalert GAPDH assay kit (Ambion). This assay measures the conversion of NAD+ to NADH when GAPDH catalyzes the oxidative phosphorylation of glyceraldehyde-3-phosphate to bisphosphoglycerate. A GAPDH standard was used construct a calibration curve for each assay done in 96-well plates. A 10μl aliquot of sample tissue lysate was added to wells of a 96-well plate in triplicate. The GAPDH reaction mix containing substrates and fluorescent dye for detection are then added and the fluorescence (530 excitation, 590 emission) immediately recorded for 5 minutes in a plate reader with automatic mixing at 30°C. Reaction rates were calculated from the change in fluorescence from the 1 to 5 minutes time interval. Units of GAPDH activity were calculated from the standard curve.

### Statistical analyses

Biochemical SOD1 expression data were presented as the mean±SEM. Kaplan-Meier and long rank were used for disease onset, paralysis and end stage using Graph Pad Prism (ver. 5.0). Rotarod data were analyzed by two-way ANOVA with Bonferoni post test.

## Results

### Changes in clinical parameters

**Fig 1:**
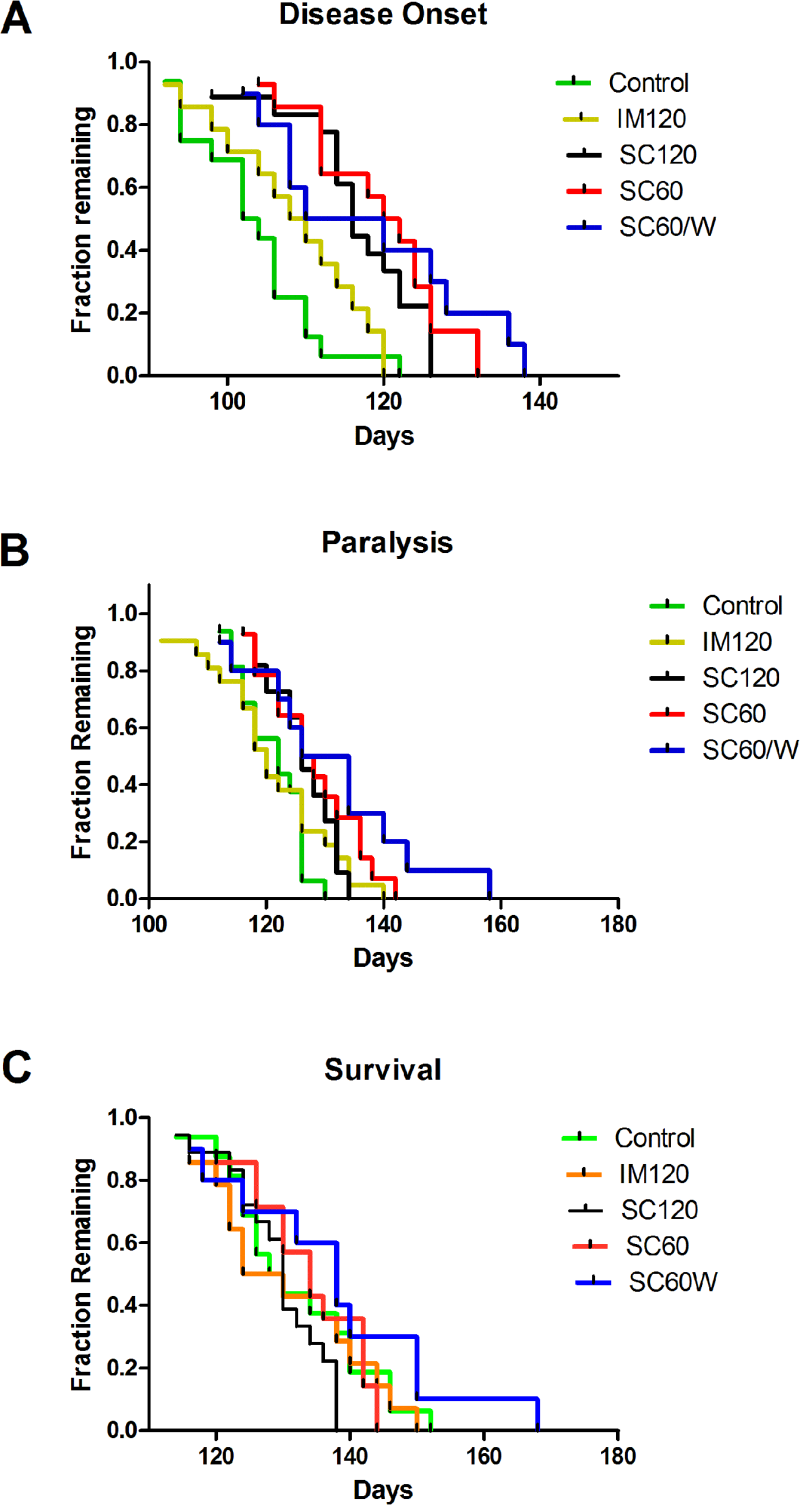
Therapeutic effects of ACTH on ALS-like symptoms in G93A-SOD1 mice. Proportion of onset (A), paralysis (B), and survival (C). Kaplan Meier was used to plot the survival curves.

Compared to the non-treated mice, ACTH treated mice showed a significant delay in disease onset with optimum effect of dose at SC60, 18 days corresponding to a 17.4% delay (Fig 1A). However the greatest delay of paralysis was seen in mice treated with SC60-W. In these mice paralysis was delayed 8 days, which is equal to 6.5% (Fig 1B). The drug did not change the survival period significantly (Fig 1C). The therapeutic effect of different ACTH doses on G93A ALS-like symptoms are summarized in Table 2.

**Table 2:** Clinical statistics of the time frame of ALS-like symptoms with corresponding treatment doses.

Animal Group	Onset/Tremor Median Age	Paralysis Median Age	Endstage Median Age
Control	103	122	129
IM120	109	120	127
SC120	116	126	130
SC60	121	127	134
SC60W	115	130	138
Log-rank significance p =	0.0001	0.0509	0.318
Log-rank trend p =	0.0001	0.0023	0.355

### Body weight and motor performance

**Fig 2:**
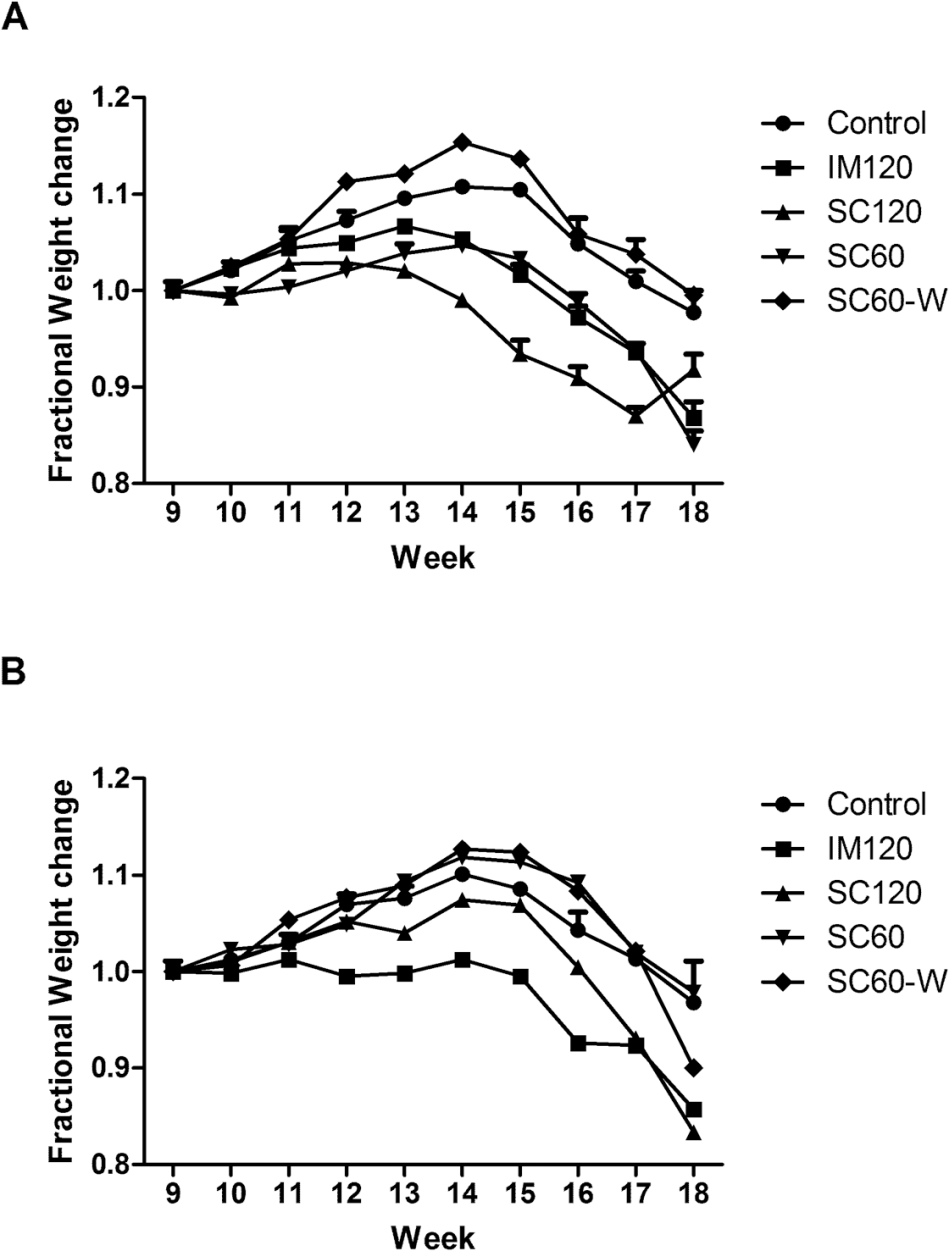
ACTH has variable dose-dependent effects on changes in the weight of G93A-SOD1 transgenic mice. Panel A shows the fractional weight change of males, while l B shows the change in female mice over the time course of treatment. Error bars are the standard deviation for each point. Bars are not visible when they are smaller than the size of the symbol.

Fractional body weight changes in both males and female G93A mice are shown in Fig 2. Panel A shows that males in the SC60-W group appeared to gain weight faster than the control and Panel B shows that SC60 and SC60-W treated female mice appeared to gain weight faster than controls. However, neither of these weight gains was significant. All other groups trended towards weight loss compared to controls that was statistically significant only for SC120 in the males (p = 0.003) and IM120 (p = 0.005) in the females. Statistical analysis of the Rotarod performance of each treated group compared to the gel-treated control group is shown in Table 3. This table shows that all drug regimens except IM120 dramatically slowed down the rotarod decline before the disease onset, with the best of male performance with SC60-W (P<0.001), and the best for females was SC60(p<0.001).

**Table 3:** Rotarod statistics.

Animal Group	Males p-value (wks)	Females p-value (wks)
IM120	> 0.05 (all)	>0.05 (all)
SC120	< 0.01 (11–13)	<0.001 (11–14)
SC60	<0.01 (10–12)	<0.001 (10–16)
SC60W	<0.001 (9–11)	<0.01 (13)

Data were analyzed by two-way ANOVA with Bonferoni post test to compare each group to control. Rotarod performances are significant for the treated groups (except for IM120) before the age of onset.

### Expression of SOD1 protein in different parts of the central nervous system

**Fig 3:**
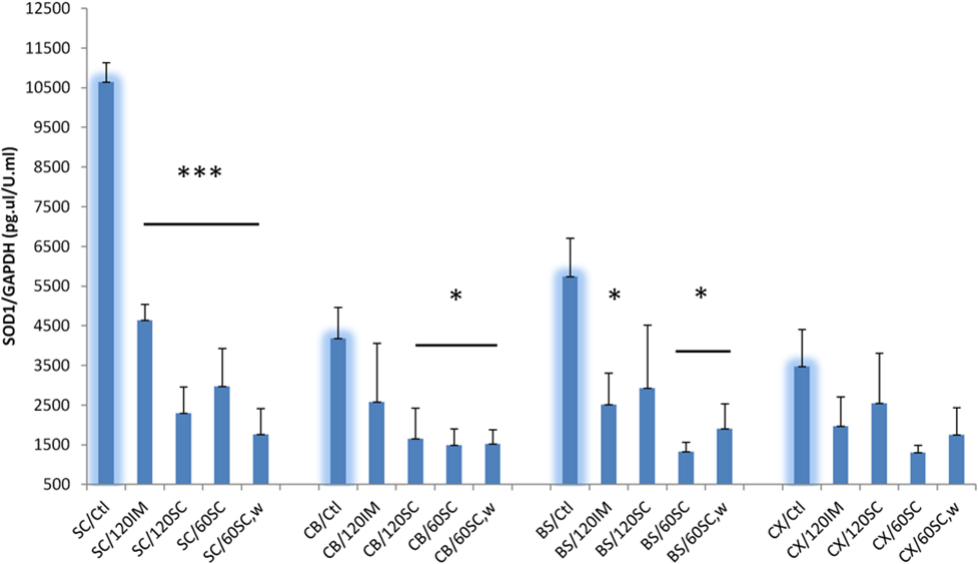
Effect of ACTH on SOD1 protein expression in different parts of the central nervous system. SOD1 was measured in the corresponding tissue lysates by ELISA and then normalized to GAPDH activity. Ctl: the nontreated animals, SC, spinal cord, CB: cerebellum, BS: brain stem, CX: cortex. n = 6 for the non-treated mice, n = 4 for treated animals. Mice treated with ACTH showed decreased expression of SOD1 protein in spinal cord and selected brain tissues.

**Fig 4:**
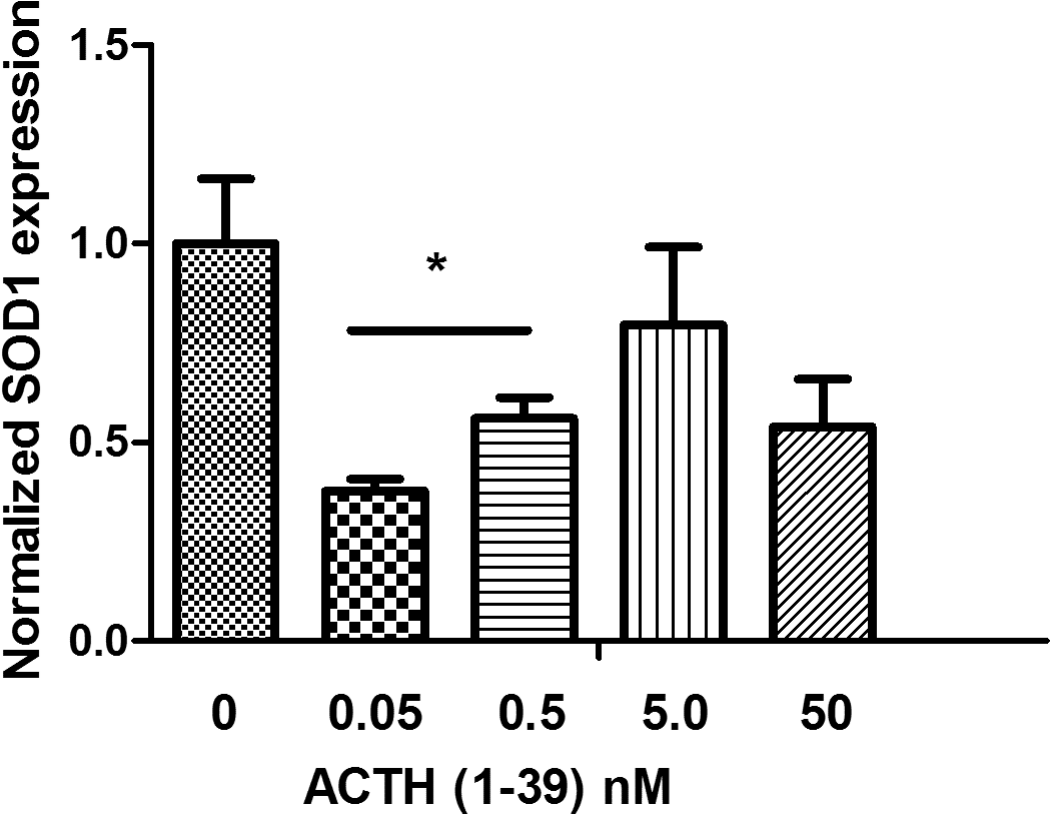
Effects of ACTH on SOD1 expression in G93A-SOD1 mouse fibroblasts. Cells were treated at the indicated concentrations of ACTH (1–39) peptide for 48 hr. Error bars indicate the standard error of the mean (n = 3, * p<0.05).

Because the onset of disease in G93A-SOD1 mice is related to the level of expression of SOD1 [25] we measured SOD1 protein levels in the central nervous system (Fig 3) of treated compared to control mice. ELISA was used to measure the soluble fraction of SOD1 protein in the corresponding tissue lysates. ACTH treatment dramatically reduced the levels of soluble SOD1 protein in the spinal cord. The reduction varied between the different drug regimens, and the optimum reduction was reached at the dose of SC60-W (84% with P<0.001). With the exception of the brain cortex, the cerebellum with dose of IM120 and the brain stem with the dose of SC120, all tested parts of the brain had significant reduction of soluble SOD1 protein by all other drug regimens. Statistically significant SOD1 reduction was dose- and tissue-dependent, ranging from 77% in the brain stem with the SC60 treatment, P = 0.007 to 57% in brain stem with the IM120 treatment, P = 0.4. It is possible that ACTH treatment reduces soluble SOD1 but increases insoluble SOD1 in mouse tissues. End stage G93A-SOD1 mice also have aggregated/particulate SOD1 in the spinal cord and brain stem that is resistant to extraction by non-denaturing detergents [41]. Therefore, we investigated the ability of ACTH (1–39) peptide to decrease SOD1 in cultured fibroblasts obtained from G93A-SOD1 mice. As shown in Fig 4, ACTH (1–39) reduces SOD1protein within the picomolar concentration range, consistent with the published data on ACTH receptor activation [42,43]. However, there is a trend towards increasing SOD1 at higher (nanomolar) concentrations of ACTH. We have observed this type of biphasic behavior previously with several types of small molecule drugs [40].

## Discussion

In this study we show that Acthar treatment has therapeutic effects on G93A mouse model of FALS, where the onset of clinical symptoms were delayed with the most effective dose by 17.4%. Paralysis showed less delay (6.5%), but was still significant. The mice treated with SC60W showed increased survival but this increase was not statistically significant, possibly due to the small number of treated mice in that group (n = 10). At the onset of disease G93A-SOD1 mice exhibit weight loss [38]. Treatment with Acthar gel slowed weight decline especially during the post-symptomatic period, with a stronger effect in females. All Acthar gel regimens (except IM120) significantly increased motor performance for a week or more just before disease onset. A surprising and unexpected finding with clear therapeutic potential was the evidence that reduction of SOD1 protein expression by Acthar is greatest in the spinal cord and brain stem of end-stage G93A mice which are the most clinically relevant regions of the CNS with respect to ALS, and to a lesser degree in other areas of the brain. Whether a decrease in SOD1protein expression occurs in other tissues or is specific to certain cell types was not examined. In preliminary studies with a different SOD1 mouse model we found that after two weeks of ACTH treatment there was not a significant reduction of SOD1 expression in the brain and spinal cord. Therefore, the effect of ACTH on SOD1 expression may be a cumulative effect requiring a longer term regiment. This may explain why ACTH did not significantly change in survival of the treated animals. Delayed therapeutic effects, such as the reduction of inflammatory cytokine production in G93A-SOD1 mice have been noted in animals treated with Erythropoietin which like ACTHar delayed disease onset and progression. [44].

Neuroinflammation occurs in ALS and other neurodegenerative diseases [45–47] and controlling neuroinflammation has been suggested as a therapeutic strategy [48]. Relatedly, ALS has also been suggested to have an autoimmune component that adversely affects neuronal cells [49] Acthar gel has been used as both an anti-inflammatory agent (idiopathic membranous nephropathy [50]) and anti-immune response agent (multiple sclerosis) with varying degrees of success [51,52]. With these precedents, we suggest that ACTH likely acts through multiple effectors within the CNS.

The novelty of this study has three features. We used a range of doses and two different routes of administration, where most of previous trials have used one dose and one route of administration of ACTH. Secondly, in contrast to humans, ACTH in rodents predominantly stimulates corticosterone over cortisol [53], so the G93A-SOD1 transgenic mouse is an ideal model to test non-cortisol mediated ACTH effects on CNS and muscle. Thirdly, with the promise of a longer regimen of therapeutic application, we have determined that SOD1protein levels are altered in the CNS including the spinal cord. In studies of changes of gene expression induced by ACTH in adrenal cells, SOD1 was not differentially expressed [54]. Therefore, testing for changes in SOD1 protein levels in other tissues may give a more complete picture of the drug’s pharmaco-kinetics, blood brain barrier penetrance, and mechanism.

This initial study has shown that the best clinical results were obtained in doses SC60, and SC60W. The latter is of interest because ACTH is also used in a pulsed fashion in the treatment of other human conditions such as multiple sclerosis and West syndrome (Infantile spasms) [51]. Further investigation is needed to determine whether lower doses or different times and route of administration are more effective.

The limitations of these studies center on the severity of disease in the G93A-SOD1 mice. Even though the survival extension in G93A-SOD1 mice was not statistically significant, it is apparent that SC60W trends to delay the disease end stage while having beneficial effects on weight and motor performance. Further optimization of dose will likely be necessary for clinical applications. Similarly, it should be noted that the effective use of Acthar gel in ALS may be limited to familial cases with SOD1 mutations. Further testing in other genetically linked disease models may be needed to expand treatment to other cases. The cortisol-stimulating effect of ACTH may be problematic in human patients, but this may be overcome by using one of several cortisol antagonists currently used for management of Cushing’s syndrome [55].
